# A Parametric Study Delineating Irreversible Electroporation from Thermal Damage Based on a Minimally Invasive Intracranial Procedure

**DOI:** 10.1186/1475-925X-10-34

**Published:** 2011-04-30

**Authors:** Paulo A Garcia, John H Rossmeisl, Robert E Neal, Thomas L Ellis, Rafael V Davalos

**Affiliations:** 1 Bioelectromechanical Systems Laboratory, School of Biomedical Engineering and Sciences, Virginia Tech - Wake Forest University, Blacksburg, VA, USA; 2 Virginia-Maryland Regional College of Veterinary Medicine, Virginia Tech, Blacksburg, VA USA; 3 Department of Neurosurgery, Wake Forest University School of Medicine, Winston-Salem, NC USA

## Abstract

**Background:**

Irreversible electroporation (IRE) is a new minimally invasive technique to kill undesirable tissue in a non-thermal manner. In order to maximize the benefits from an IRE procedure, the pulse parameters and electrode configuration must be optimized to achieve complete coverage of the targeted tissue while preventing thermal damage due to excessive Joule heating.

**Methods:**

We developed numerical simulations of typical protocols based on a previously published computed tomographic (CT) guided *in vivo *procedure. These models were adapted to assess the effects of temperature, electroporation, pulse duration, and repetition rate on the volumes of tissue undergoing IRE alone or in superposition with thermal damage.

**Results:**

Nine different combinations of voltage and pulse frequency were investigated, five of which resulted in IRE alone while four produced IRE in superposition with thermal damage.

**Conclusions:**

The parametric study evaluated the influence of pulse frequency and applied voltage on treatment volumes, and refined a proposed method to delineate IRE from thermal damage. We confirm that determining an IRE treatment protocol requires incorporating all the physical effects of electroporation, and that these effects may have significant implications in treatment planning and outcome assessment. The goal of the manuscript is to provide the reader with the numerical methods to assess multiple-pulse electroporation treatment protocols in order to isolate IRE from thermal damage and capitalize on the benefits of a non-thermal mode of tissue ablation.

## Background

Irreversible electroporation is a new technique for the focal ablation of undesirable tissue using high voltage, low energy electric pulses [1,2]. An IRE treatment involves placing electrodes within the region of interest and delivering a series of electric pulses that are microseconds in duration [3]. The pulses create an electric field that induces an increase in the resting transmembrane potential (TMP) of the cells in the tissue [4]. The induced increase in the TMP is dependent on the electric pulse (e.g. strength, duration, repetition rate, shape, and number) and physical configuration of the electrodes used to deliver the pulses. Depending on the magnitude of the induced TMP, as well as its duration and repetition rate for induction, the electric pulses can have no effect, transiently increase membrane permeability, or cause cell death [5]. Spatially, for a given set of conditions, the TMP and therefore the degree of electroporation is dependent on the local electric field to which the cells are exposed. Because the transitions in cellular response to the electric pulses are sudden, the treated regions are sharply delineated. Consequently, numerical models that simulate the electric field distributions in tissue are needed to predict the treated region [6-8].

There have been several studies evaluating the efficacy and safety of IRE in treating both experimental and spontaneous tumors. Al-Sakere *et al. *subcutaneously implanted sarcoma tumors in mice and achieved a complete response in 12 of 13 tumors with IRE treatment [1]. Guo *et al. *achieved regression of hepatocellular carcinoma tumors implanted in liver in 9 out of 10 rats treated with IRE [9]. Neal *et al. *implanted human mammary tumors orthotopically in mice and produced a complete response in 5 of 7 tumors with IRE which verified that IRE can be used in a heterogeneous environment [10]. In a clinical series of IRE based therapies, our group has long-term follow-up on canine patients with spontaneous tumors. One canine patient was treated with IRE and radiation therapy for a non-resectable, high-grade glioma, resulting in complete remission of the tumor at four months [11]. Another canine patient with a focal histiocytic sarcoma has been in complete remission for 8 months since completion of the last IRE treatment [12].

One of the main advantages of IRE over other focal ablation techniques is that the therapy does not use thermal damage from Joule heating to kill the cells. As a result, major blood vessels, extracellular matrix and other critical structures are spared [1,2]. Because electroporation based therapies require high-voltage pulses to be administered to the tissue, thermistors and thermocouples may become damaged during treatment. Therefore, previous investigations into the thermal aspects of electroporation based therapies have relied on numerical modeling, typically using a modified Pennes' Bioheat equation with an added Joule heating term to predict the thermal effects. There have been several theoretical attempts in the literature to investigate the thermal response of tissues to electroporation-based treatments and assess the degree, if any, of thermal damage. In some studies, the authors calculate the pulse time required to reach a maximum temperature of 50°C, which they assume is when instantaneous thermal damage will occur [13,14]. Others calculate the equivalent thermal dose or thermal damage associated with one or multiple pulses to determine the amount, if any, of tissue damage due to exposure of the tissue to elevated temperatures [4,15-19]. Finally, other papers show the equivalent thermal dose for an 80-pulse IRE treatment [1]. Pliquett *et al. *performed a qualitative assessment of thermal effects induced by electroporation by using temperature-sensitive liquid crystals that change colors at 40°C, 45°C, and 50°C [20]. Although these theoretical and qualitative analyses are very powerful and well-grounded, to the best of our knowledge there is no experimental data for actual temperature changes during IRE pulse administration to prove that cell death occurs independent of classical thermal-induced mechanisms. This data is vital in order to validate the numerical models and better predict the temperature changes during a procedure for thermal damage assessment. In addition, the numerical models assessing thermal damage in the literature do not simultaneously incorporate the significant changes in the electrical conductivity of the tissue due to temperature changes as well as electroporation. Therefore, models of electroporation-based protocols that include the electrical conductivity changes and do not assume that the heat will dissipate by the beginning of the following pulse are needed in order capture the entire thermal effects of a procedure. It is then possible to maximize the sparing of critical structures in the brain and other organs and to determine the upper limit of the IRE treatment, above which thermal damage ensues. Accurate prediction of all treatment associated effects is vital to the development and implementation of optimized treatment protocols.

Our group has confirmed the safety of intracranial IRE procedures in three experimental canines [21]. These procedures were performed through craniectomy defects to expose the cortex (grey matter) and allow for the insertion of the electrodes in the brain. We have also correlated numerical models with 3D lesion reconstructions in order to establish electric field intensities needed to kill grey matter [22]. These studies have shown that IRE has the potential to treat intracranial disorders in canine and human patients. In the present study, we use a previously reported treatment performed through 1.2 mm diameter burr holes with CT guidance placement within a subcortical neuroanatomic target as the basis for a parametric study [23].

The parametric study in brain tissue evaluates the effects that the change in tissue electrical conductivity due to electroporation and the thermal effects have on the electric field distribution. It further simulates treatment volumes for similar procedures performed at three frequencies that have been used clinically in other tissues including prostate, kidney and lungs [24-27]. This study demonstrates how one can use an Arrhenius analysis to relate temperature and length of exposure during electroporation-based procedures.

## Methods

### Clinical Procedure

**Figure 1:**
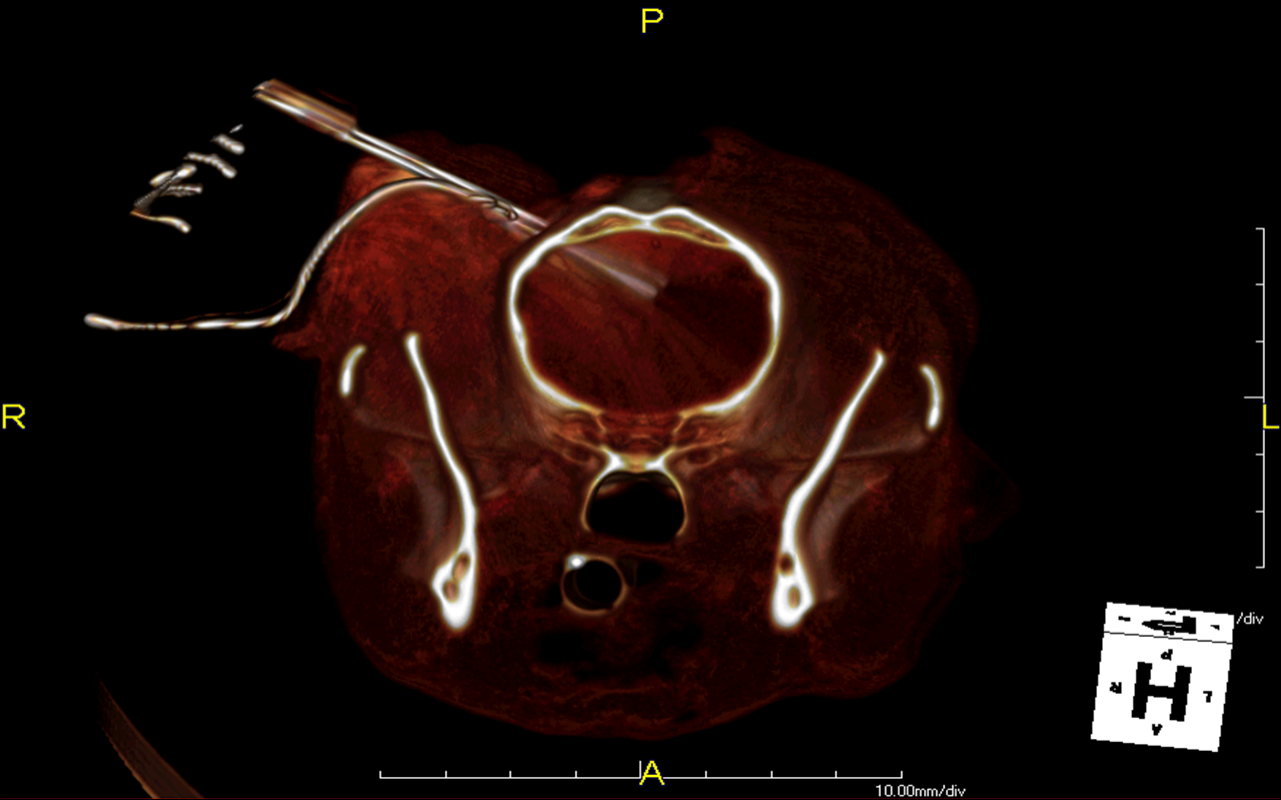
**Reconstructed computed tomographic (CT) scans confirming the placement of the electrodes**. The electrodes were placed into the targeted deep white matter of the corpus callosum of a canine prior to the delivery of the IRE pulses. The procedure was performed in a minimally invasive fashion through 1.2 mm diameter burr holes.

The experimental aspect of the study is described in detail within our previously published conference proceeding [23], and was approved by the Institutional Animal Care and Use Committee and performed in a Good Laboratory Practices (GLP) compliant facility. After induction of general anesthesia in the canine, two 1.2 mm diameter burr holes were created in the skull in preparation for electrode insertion [23]. The CT guidance system was used to place the electrodes into the targeted deep white matter of the corpus callosum, as seen in Figure 1 (TeraRecon, Foster City, CA) [23].

A neuromuscular blocker was administered to suppress patient motion prior to the IRE treatment [23]. A focal ablative IRE lesion was created in the white matter of the brain using the NanoKnife^® ^generator (Angiodynamics, Queensbury, NY USA) [23]. Two 1-mm diameter blunt tip electrodes with 5 mm exposure were inserted into the brain through the burr holes with a center-to-center separation distance of 5 mm [23]. After insertion of the electrodes, four sets of twenty, 50 μs long, electric pulses were delivered with an applied voltage of 500 V [23]. The polarity of the electrodes was alternated between the sets to minimize charge build-up on the electrode surface [23]. These parameters were determined from our previous *in vivo *intracranial IRE procedures which showed that they were sufficient to ablate grey matter [21,22,28]. The NanoKnife^® ^pulses were synchronized with the canine's heart rate in order to prevent ventricular fibrillation or other cardiac arrhythmias and were delivered in trains of ten [23]. Due to recharging demands of the capacitors, each train of ten pulses was delivered 3.5 seconds after completion of the previous train.

#### Temperature Measurements

**Figure 2:**
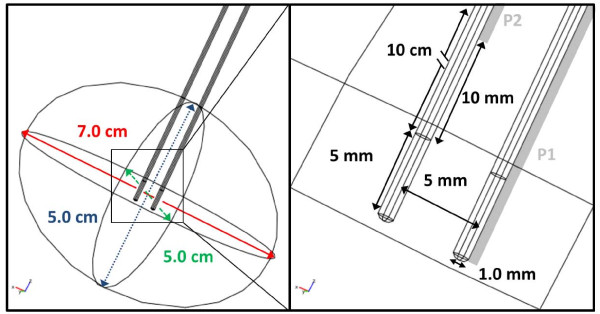
**Schematic of the brain and electrode configuration used in the numerical models**. The thermal probes used to measure the temperature during the experimental procedure are shown in light gray on the right panel. Probe 1 (P1) and probe 2 (P2) were located near the electrode tip and 10 mm along the insulation of the other electrode, respectively.

Temperatures were measured in the brain during the procedure using the Luxtron^® ^m3300 Biomedical Lab Kit Fluoroptic^® ^Thermometer and STB medical fiber optic probes (LumaSense™ Technologies, Santa Clara, CA USA). The probes, which are immune to electromagnetic interference, consist of a fiber optic cable terminated with a temperature sensitive phosphorescent sensor. Pulsed light strikes the phosphorescent element causing it to fluoresce. The decay time of this fluorescent signal is temperature dependent and is measured with an accuracy of ± 0.2°C. In order to minimize the invasiveness of the procedure, the thermal probes were placed within a 0.78 mm outer diameter polyimide tubing that was attached near the tip of the electrode-tissue interface and 10 mm along the insulation as seen in Figure 2[23]. The data acquisition was performed with TrueTemp™ software (Version 2.0, Luxtron^® ^Corporation, Santa Clara, CA USA) in which each probe was set to a recording frequency of 2 Hz. The measured temperature was imported into Wolfram Mathematica 6.0 for students (Champaign, IL USA) for analysis. The oscillatory data was smoothed with the moving average command in which each data point reported is the average of the neighboring ± 10 data points. We present the raw and the smoothed versions of the temperature data in the results section.

#### Image Acquisition

Immediately after completion of the pulses, the subject was imaged with CT and a 0.2 T magnetic resonance imaging (MRI) system. The animal was then humanely euthanized 2 hours post-IRE by intravenous barbiturates and the brain was harvested and fixed in 10% buffered formalin. The fixed *ex vivo *brain was later imaged on a 7.0 T MRI for a more detailed analysis of the lesion produced.

### Numerical Models

Numerical models can be used for treatment planning to ensure that only the targeted regions are ablated [8]. In order for this to be accurate, one has to know both the physical properties of the tissue, the electric field distribution, and the electric field threshold needed for IRE. This study examined two sets of models. The first was developed to replicate the experimental procedure and used the temperature and current data to calibrate the properties and behavior of the tissue in response to the electric pulses. After calibrating the model with properties based on the experimental procedure, the model was adjusted to simulate treatments at three pulse repetition rates (0.5, 1, and 4 Hz) and three voltages (500, 1000, and 1500 V) for up to 80 pulses. The computations were performed with a commercial finite element package (Comsol Multiphysics, v.3.5a, Stockholm, Sweden).

#### Electric Field Distribution

**Figure 3:**
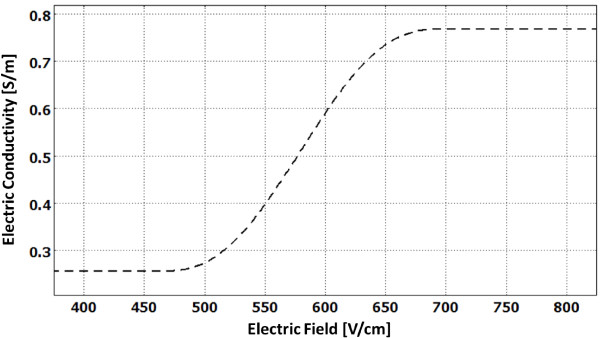
**Electric conductivity of tissue as a function of the local electric field**. This plot was determined by the flc2hs Heaviside function. The conductivity changes from a baseline σ_0 _= 0.256 S/m to σ = 3.0 · σ_0 _= 0.767 S/m at the onset of the IRE pulses. Note: We assumed that once the conductivity increased due to electroporation it would not revert back.

The methods used to generate the electric field and temperature distributions in tissue are similar to the ones described by several investigators [4,6-8,22]. The electric field distribution associated with the electric pulse is given by solving the governing Laplace equation:(1)

where σ is the electrical conductivity of the tissue and *φ *is the electrical potential [8]. The baseline electrical conductivity of the non-permeabilized white matter, *σ*_0 _= 0.256 *S*/*m*, was based on measurents by Latikka *et al. *in living humans at 37°C [29]. However, a tissue's conductivity is also a function of its temperature and any electropermeabilization induced by the electric pulses [30-33]. Therefore, the electrical conductivity was modeled dynamically to incorporate changes due to electroporation and thermal effects and is described by(2)

where *σ*_0 _is the baseline conductivity, *α *the temperature coefficient, *T *the temperature, and *T*_0 _the physiological temperature [22]. Figure 3 displays the smoothed Heaviside function, *flc*2*hs*, with a continuous second derivative that ensures convergence of the numerical solution. This function is defined in Comsol, and it changes from zero to one when *normE*_*dc *- *E_delta _*= 0 over the range ± *E_range _*[22].

Initially, the 3D simulation was solved for a negligible fraction of the total treatment duration under homogenous tissue conditions in order to establish a baseline electric field distribution. The homogeneous electric field map provides the starting values for the dynamic conductivity function [22]. In our function, we assumed that the conductivity would increase by a factor of 3.0 due to electroporation, since this is similar to the reported factor in other organs during electroporation [30,33-37]. Additionally, this factor matches the experimental current (data not shown) that was measured by the NanoKnife^® ^after the transient membrane charging effects had settled during the delivery of the pulses [23].

The brain was modeled as a 7.0 cm × 5.0 cm × 5.0 cm ellipsoid with the electrodes inserted to a maximum depth of 2.5 cm (Figure 2). Because electrode placement resulted in the electrodes being surrounded mainly by white matter, homogeneous physical properties were set to those of white matter. The electrodes were modeled as an insulating body with an extension of stainless steel. Boundary conditions often include surfaces where electric potential is specified, as in the case of a source or sink electrode, or surfaces that are electrically insulating, as on the free surfaces of the tissue, for example. The electrical boundary condition along the tissue that is in contact with the energized electrode was *φ *= V_o_. The electrical boundary condition at the interface of the other electrode was *φ *= 0. The remaining boundaries were treated as electrically insulating:(3)

The models are fully defined and readily solvable using a numerical method once an appropriate set of boundary conditions and the properties of the tissue are defined (Table 1).

**Table 1:** Physical properties used in the numerical simulations.

MATERIAL	QUANTITY	UNITS	VALUE	REFERENCE
Brain	α, temperature coefficient	°C^-1^	0.032	[39]
	k, thermal conductivity	W m^-1 ^K^-1^	0.565	[39]
	C_p _, heat capacity	J kg^-1 ^K^-1^	3680	[39]
	ρ, density	kg m^-3^	1039	[39]
	q''', metabolic heat generation	W m^-3^	10437	[40]

Blood	C_b _, heat capacity	J kg^-1 ^K^-1^	3840	[40]
	ρ_b _, density	kg m^-3^	1060	[41]
	w_b _, perfusion rate	s^-1^	7.15E-3	[41]

Insulation	σ, electrical conductivity	S m^-1^	1.0E-5	[42]
	k, thermal conductivity	W m^-1 ^K^-1^	0.01	[42]
	C_p _, heat capacity	J kg^-1 ^K^-1^	3400	[42]
	ρ, density	kg m^-3^	800	[42]

Stainless Steel	σ, electrical conductivity	S m^-1^	2.22E6	[1]
	k, thermal conductivity	_W m_^-1 ^_K_^-1^	15	[42]
	C_p _, heat capacity	J kg^-1 ^K^-1^	500	[42]
	ρ, density	kg m^-3^	7900	[42]

Instead of modeling eighty individual pulses, we modified the approach to have a continuous delivery of the electric field since we assume that once the conductivity increased due to electroporation it would not revert back. Using this approach eliminates the need to manipulate the time steps in order to ensure that the microsecond pulses are captured by the solver. This helps the simulation run faster and smoother since there are no abrupt changes due to the pulses. In order to deliver the same amount of energy as in the pulsed approach, we multiplied the Joule heating by the duty cycle (duration/period) of the pulse in the tissue and insulation domains. This ensures that at the onset of each pulse, equal amounts of thermal energy have been deposited in the tissue using either approach.

#### Temperature Distribution

The Pennes' Bioheat equation is often used to assess tissue heating associated with thermally relevant procedures, because it accounts for the dynamic processes that occur in tissues, such as blood perfusion and metabolism. Blood perfusion is an effective way to dissipate heat in contrast to metabolic processes which generate heat in the tissue. Modifying this equation to include the Joule heating term gives the equation the following form:(4)

where *k *is the thermal conductivity of the tissue, *T *is the temperature above the arterial temperature (*T_a _*= 37°*C*), *w_b _*is the blood perfusion rate, *C_b _*is the heat capacity of the blood, *ρ_b _*the blood density, *q''' *is the metabolic heat generation, *ρ *is the tissue density, and *C_p _*is the heat capacity of the tissue. Several thermal boundary conditions can be employed to study the heat exchange between the electrodes and the tissue [13,17,38]. In our models, the electrodes were considered as heat sinks, , which dissipate heat from the tissue through the electrodes to the environment [19,22].

#### Thermal Damage Distribution

Thermal damage occurs when tissues are exposed to temperatures higher than their physiological temperature for extended periods of time. If the period of exposure is long, thermal damage can occur at temperatures as low as 43°C, while 50°C is generally chosen as the target temperature for instantaneous thermal damage [43]. This damage can represent a variety of processes including cell death, microvascular blood flow stasis and/or protein coagulation [44]. The thermal effects can be calculated to assess whether a particular set of pulse parameters and electrode configuration will induce thermal damage in superposition with IRE. The damage can be quantified using an Arrhenius type analysis which assumes that the damage follows first order reaction kinetics given by:(5)

where *ζ *is the frequency factor,*E_a _*the activation energy, *R *the universal gas constant, *T*(*t*) is the temperature distribution and *τ *is the heating time [4,15,44,45]. It has been shown that Ω = 0.53 is the threshold for burn injuries in blood-perfused skin [45-47]. We have adapted the Arrhenius equation, which traditionally has been used to study burn injuries in skin and transdermal drug delivery using electroporation, to investigate therapeutic IRE.

In order to compute if any thermal damage resulted from the procedure, a time-dependent analysis partial differential equation (PDE) was added under the PDE Mode in Comsol Multiphysics to simultaneously solve the distributions of the electrical potential, temperature, and thermal damage within the domain. The temperatures were calculated with the modified Pennes' Bioheat equation described above. Thermal damage was computed in the entire tissue domain in order to perform a comprehensive analysis of the thermal effects. The expression to calculate the damage is given by(6)

where Ω is the damage, the Γ is the flux vector and *F *is the forcing function(7)

The forcing function is written in logarithmic form in order to prevent abrupt changes in the solver since small changes in temperature can have significant impact on the damage. The flux vector was assumed to be zero since heat conduction is already incorporated in Equation 4. Similarly, all the boundaries in the domain were assumed to be of the Neumann form where . The analysis was performed with a starting temperature equal to physiological conditions and the cell death parameters from Table 2.

**Table 2:** Activation energy (*E*_*a*_) and frequency factor (*ζ*) for thermal damage processes [44].

DAMAGE PROCESS	*E_a _*[J mol^-1^]	*ζ *[s^-1^]	REFERENCE
Microvascular Blood Flow Stasis	6.670 × 10^5^	1.98 × 10^106^	[48]
Cell Death	5.064 × 10^5^	2.984 × 10^80^	[49]
Protein Coagulation	2.577 × 10^5^	7.39 × 10^37^	[50]

## Results

### Clinical Procedure

**Figure 4:**
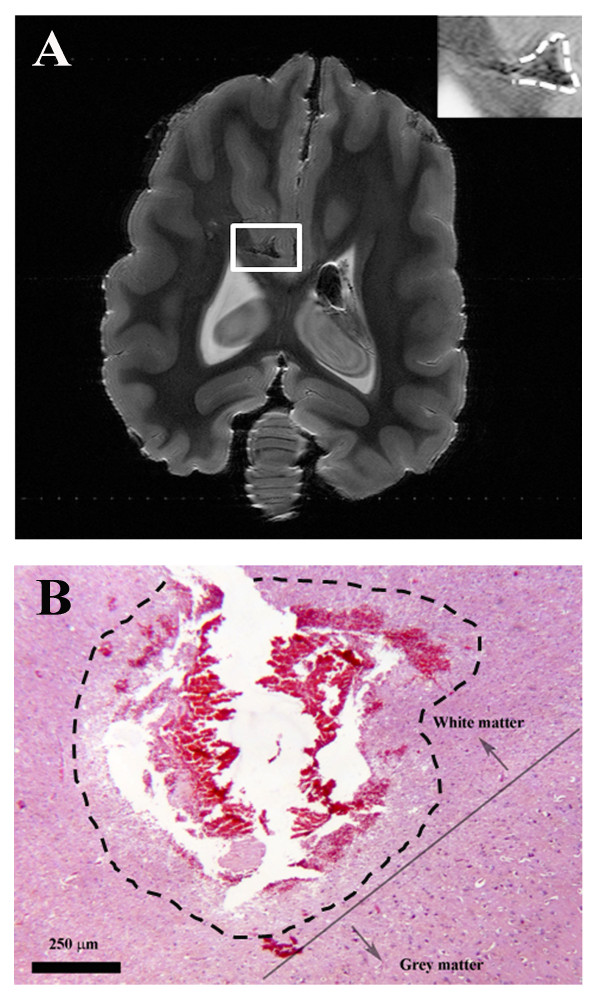
**Focal and cavitary white matter areas of ablation induced by IRE**. The lesion is illustrated with dashed lines using (A) ex vivo 7.0 T MRI in the dorsal plane (insert) and (B) histopathology, hematoxylin and eosin stain.

The 0.2 T MRI showed a focal, well circumscribed IRE lesion with calculated volumes of 0.131 cm^3 ^and 0.120 cm^3 ^for the T1-weigthed post-contrast and T2-weighted MRIs, respectively which we reported in Garcia *et. al *[23]. The lesion appeared hyperintense within the white matter on the T1-weighted post-contrast MRI, where contrast was able to leak into the brain due to breakdown of the blood-brain-barrier. The lesion was also hyperintense on the T2-weighted MRI sequence. Figure 4 demonstrates the focal and cavitary nature of the ablative white matter lesion within 2 hours after pulsing on both the *ex vivo *7.0 T MRI (Figure 4A) and with light microscopy (Figure 4B). The most affected region appears to be directly between the electrodes, which is where the highest electric fields were generated. The reconstructed lesion volume from the high-resolution 7.0 T MRI was 0.058 cm^3 ^[23].

#### Experimental Temperature Distribution

**Figure 5:**
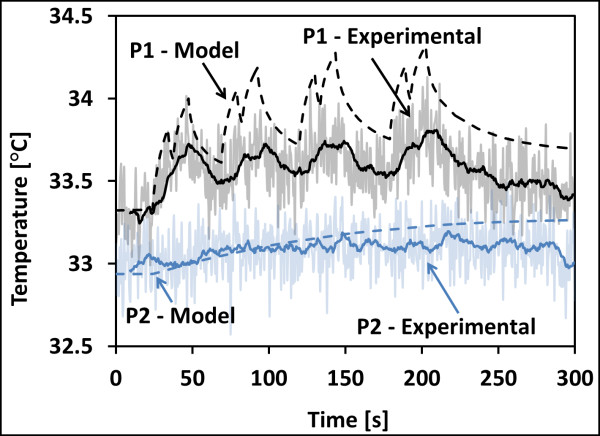
**Temperatures measured and calculated during an IRE treatment in white matter of brain**. Four sets of twenty 50 μs pulses with and applied voltage of 500 V were delivered at approximately 1 Hz. The measured temperatures are shown with solid lines and the calculated temperatures with dashed lines.

Figure 5 shows the raw and smoothed experimental temperature (solid) distributions measured with the thermal probes near the tip of the electrode-tissue interface and 10 mm above the insulation [23]. For the probe at the electrode-tissue interface (P1), four sets of mild increases in temperature are seen, which corresponds to each of the pulse sets delivered. The probe at the insulation (P2) shows minimal increase in temperature, mostly appearing due to heat conduction from the treatment region. The experimental changes in temperature resulting from the pulses were less than 1.15°C and were insufficient to generate thermal damage. This confirms that any cell death achieved by the procedure was a direct result of IRE since numerical simulations near the electrode-tissue interface routinely experience the greatest thermal effects [8,14,51]. It is important to note that the starting temperature was approximately 33°C due to the anesthesia effects. However, a starting temperature of 37°C was used in the numerical models investigated in the parametric study.

Figure 5 also includes the calculated temperature (dashed) distribution from the calibration model at the two locations where the thermal probes were positioned experimentally. This numerical simulation replicated all aspects of the experimental procedure including the four sets of twenty pulses and the 3.5 seconds delay after the first ten pulses in each set due to the recharging demands of the capacitors. Even though the starting temperature was set to 33°C, we scaled the resulting initial temperatures to match the experimental values in order to provide a more objective comparison. From this figure, it is clear that the temperatures calculated with the numerical model were marginally higher than the measured ones. This calibration model was used as the basis for the parametric study since we were able to closely match the experimental and calculated temperature and electrical current.

### Numerical Models

**Figure 6:**
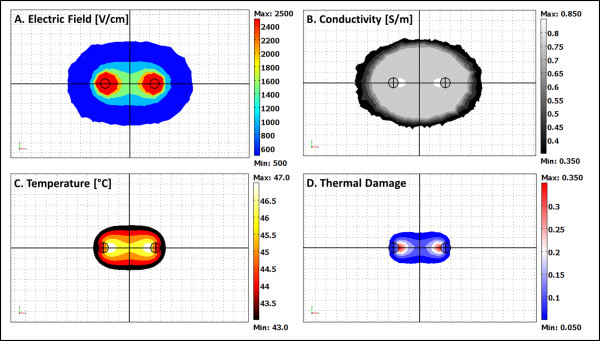
**Electric field, conductivity, temperature and thermal damage distributions at the conclusion of an 80 s IRE treatment simulation**. A model of eighty 50 μs pulses with an applied voltage of 1000 V at a repetition rate of 1 Hz is presented. The grid resolution in the distributions is 1.0 mm. For this specific simulation, the tissue was treated only with IRE since thermal damage occurs when Ω > 0.53.

Figure 6 is a representation of the results from the simulated IRE treatments in brain. This figure displays the electric field, conductivity, temperature, and thermal damage distributions at the end of an entire IRE protocol. The electric field and temperature distributions are critical since they allow for the numerical integration of the electric field (Figure 6A) to determine volumes of IRE and temperature (Figure 6C) to assess thermal effects including thermal damage, respectively (Figure 6D). We provide these distributions for one time point (80 s) and treatment parameter set (e.g. eighty 50-μs pulses at 1000 V delivered at 1 Hz), but could readily report any of the other simulated protocols. Figure 6A displays the electric field distribution on the tissue treated with IRE. Figure 6B shows the distribution of the electrical conductivity of the tissue as given by Equation 2. Figure 6C presents the temperatures at the completion of the pulse delivery. Figure 6D uses the temperature data throughout the treatment delivery to assess the presence of thermal damage. The maximum temperature reached was 47.8°C, with a thermal damage value, Ω, of 0.38. The increase in temperature during this simulation did not generate any tissue death by thermal modes since Ω was below the 0.53 threshold needed for thermal damage.

#### Parametric Study Model

After creating and calibrating the numerical model to the experimental data, a parametric study was performed to analyze the effects of varying the IRE treatment by using three pulse repetition rates (0.5, 1, and 4 Hz) and three voltages (500, 1000, and 1500 V) for up to 80 pulses. From these models, the volume of tissue treated by IRE as well as temperature changes and thermal damage was analyzed. Table 3 tabulates the calculated volumes of tissue that were treated with IRE at the onset and completion of the eighty pulses for all treatment scenarios considered, and also compares predictions drawn from the models using static conductivity and the dynamic conductivity equation. Furthermore, the time history of each volumetric quantity for IRE and the thermal assessments are presented to provide a clear delineation of treatment protocols that achieve IRE alone or in superposition with thermal damage.

**Table 3:** Volumes (cm^3^) of tissue treated with IRE for static *σ*(*T*) and dynamic *σ*(*E, T*) conductivities.

VOLTAGE (V)		*σ*(*T*)_*t *= 0_	*σ*(*E, T*)_*t *= 0_				
**0.5 Hz**	**500**	0.179	0.291	0.630	0.179	0.293	0.635
	**1000**	0.418	0.695	0.665	0.424	0.706	0.665
	**1500**	0.654	1.103	0.686	0.683	1.134	0.660

**1 Hz**	**500**	0.179	0.291	0.630	0.179	0.293	0.633
	**1000**	0.418	0.695	0.665	0.430	0.710	0.652
	**1500**	0.655	1.103	0.683	0.710	1.158	0.630

**4 Hz**	**500**	0.179	0.291	0.629	0.182	0.293	0.613
	**1000**	0.418	0.695	0.665	0.460	0.732	0.591
	**1500**	0.656	1.103	0.681	0.835	1.296	0.553

We report the volumes of tissue treated with IRE as those exposed to a minimum electric field of 500 V/cm, which was found to be the IRE threshold in grey matter for similar pulse parameters to those used in this parametric study [22]. Although we used a 500 V/cm threshold for our calculations, other researchers could adapt the numerical model to investigate the results of IRE in other tissues where the threshold could be different. In order to provide insight to the reader, we modeled the electrical conductivity of the tissue with static and dynamic functions. In the static function, ***σ***(***T***), the electrical conductivity of the tissue was assumed to be homogeneous and dependent only on the temperature. The dynamic function, ***σ***(***E*, *T***), incorporated the dependency of the electrical conductivity on temperature and electroporation. Applying 500 V at 0.5, 1, and 4 Hz resulted in IRE treated volumes between 0.179 - 0.182 cm^3 ^for the static function and 0.293 cm^3 ^for the dynamic function. The IRE treated volumes ranged between 0.424 - 0.460 cm^3 ^for the static function and between 0.706 - 0.732 cm^3 ^for the dynamic function when the applied voltage was 1000 V. Finally, applying 1500 V generated IRE volumes between 0.683 - 0.835 cm^3 ^for the static models and between 1.134 - 1.296 cm^3 ^for the dynamic ones. The IRE treatment volume increased 55% - 69% when the dynamic conductivity function was incorporated as compared to the static conductivity function. The results show the importance of using a conductivity function that takes into account all the relevant physical phenomena that occurs during electroporation in order to provide accurate treatment planning [22,30-32]. Researchers and physicians should be aware of the increase in treatment volumes due to electroporation and temperature based conductivity changes when performing treatment planning as has been described by several groups in the field [21,30-33]. Other groups have developed algorithms that are capable of determining optimum electrode configuration and optimum amplitude of the electric pulses for treatment planning of electroporation-based therapies [52,53].

#### Electrical Current Distribution

**Figure 7:**
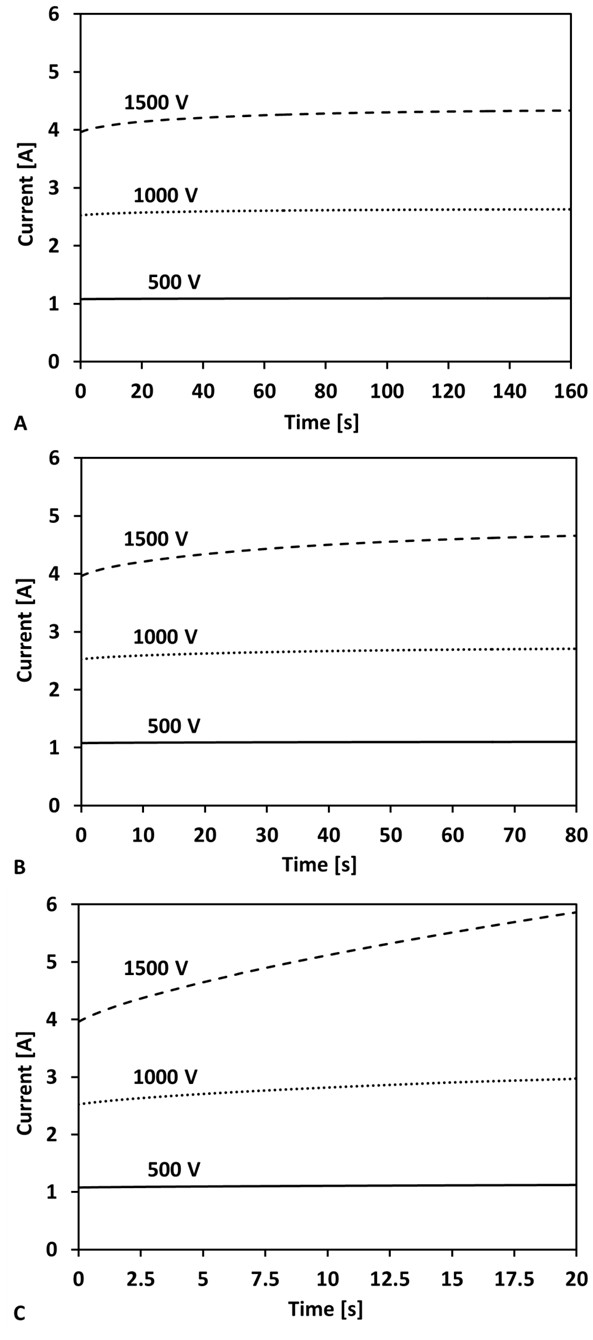
**Time history of the total current during an eighty pulse (50 μs) IRE treatment**. The pulses were delivered at frequencies of A) 0.5 Hz (160 s), B) 1 Hz (80 s), and C) 4 Hz (20 s). The applied voltages were 500 V, 1000 V, and 1500 V for each frequency investigated.

In addition to monitoring the temperature during the experimental procedure, the current of each individual pulse was measured by the NanoKnife^®^. It was found that the current throughout the procedure was 1.11 ± 0.2 A [23]. The resulting currents from the parametric IRE simulations for the 500, 1000, and 1500 V treatments delivered at 0.5, 1, and 4 Hz were calculated and are displayed in Figure 7. Applying 500 V resulted in electrical currents of 1.08 - 1.12 A independent of the pulse repetition rate. A current ranging between 2.53 - 2.98 A was calculated when using 1000 V. Simulations at 1500 V resulted in an initial current of 3.96 A, and reached 4.33 A (0.5 Hz), 4.66 A (1 Hz), or 5.90 A (4 Hz) at the completion of the pulses. The variation between the calculated currents can be explained by the increase in temperature during the pulse delivery. When the pulses were delivered at 4 Hz, there was less time for the heat to dissipate through conduction or blood perfusion. Therefore, the calculated temperatures were significantly higher which also resulted in higher electrical conductivity and thus electrical current. The increase in electrical current was not observed during the 500 V treatments since at this lower voltage the thermal effects are negligible compared to the changes in conductivity due to electroporation. The measured current agreed with the calculated currents, validating our assumption of an increase in the brain electric conductivity by a factor of 3.0 due to electroporation.

### Irreversible Electroporation v. Thermal Effects

#### Traditional Thermal Assessment

**Figure 8:**
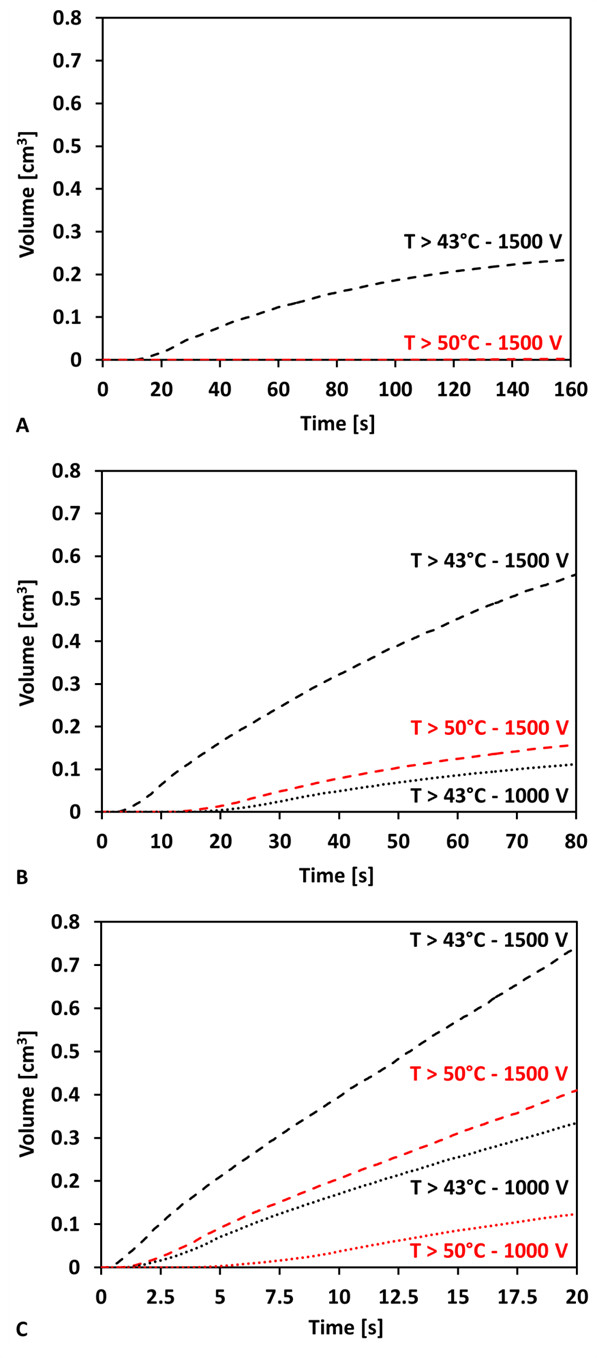
**Time history of the volumes of tissue exposed to temperatures greater than 43°C and 50°C**. The IRE treatment used eighty pulses (50 μs) with pulse frequencies of A) 0.5 Hz (160 s), B) 1 Hz (80 s), and C) 4 Hz (20 s). The applied voltages were 500 V, 1000 V, and 1500 V for each frequency investigated.

The volumes of tissue presented in this section were used to calculate the percentage of the tissue that was treated with IRE in superposition with the thermal assessment and are given in parentheses. The curves in Figure 8 are calculated volumes of tissue exposed to temperatures greater than 43°C and 50°C. These values have been used for the assessment of potentially thermally damaging temperatures with 43°C being used for extended exposures and 50°C for instantaneous thermal damage [4]. Figure 8A shows that at the completion of the treatments using a 0.5 Hz pulse repetition rate, volumes of tissue exposed to temperatures greater than 43°C and 50°C were only achieved when delivering 1500 V, up to maximum volumes of 0.235 cm^3 ^(20.7% - 43°C) and 0.002 cm^3 ^(0.2% - 50°C). However, the effects of temperature become more significant when the pulses are delivered at a higher repetition rate, shown in Figure 8B for a frequency of 1 Hz (80 s for total treatment). Here, applying 1000 V resulted in 0.112 cm^3 ^(15.8%) of the tissue exposed to temperatures greater than 43°C and 0.00 cm^3 ^(0.0%) at 50°C, significantly lower than the 1500 V treatment, which had tissue volumes of 0.557 cm^3 ^(48.1%) and 0.158 cm^3 ^(13.7%) exposed to temperatures greater than 43°C and 50°C, respectively. In Figure 8C one can appreciate the drastic effects of further increasing the repetition rate to 4 Hz (20 s for total treatment). In this scenario, even 1000 V results in tissue heating above 50°C in 0.124 cm^3 ^(16.9%) of tissue, and greater than 43°C in 0.335 cm^3 ^(45.7%). Finally, for an applied voltage of 1500 V, the majority of the tissue will be heated to elevated temperatures, where 0.741 cm^3 ^(57.2%) and 0.410 cm^3 ^(31.7%) of tissue experiences temperatures greater than 43°C and 50°C, respectively.

#### Thermal Damage Assessment

**Figure 9:**
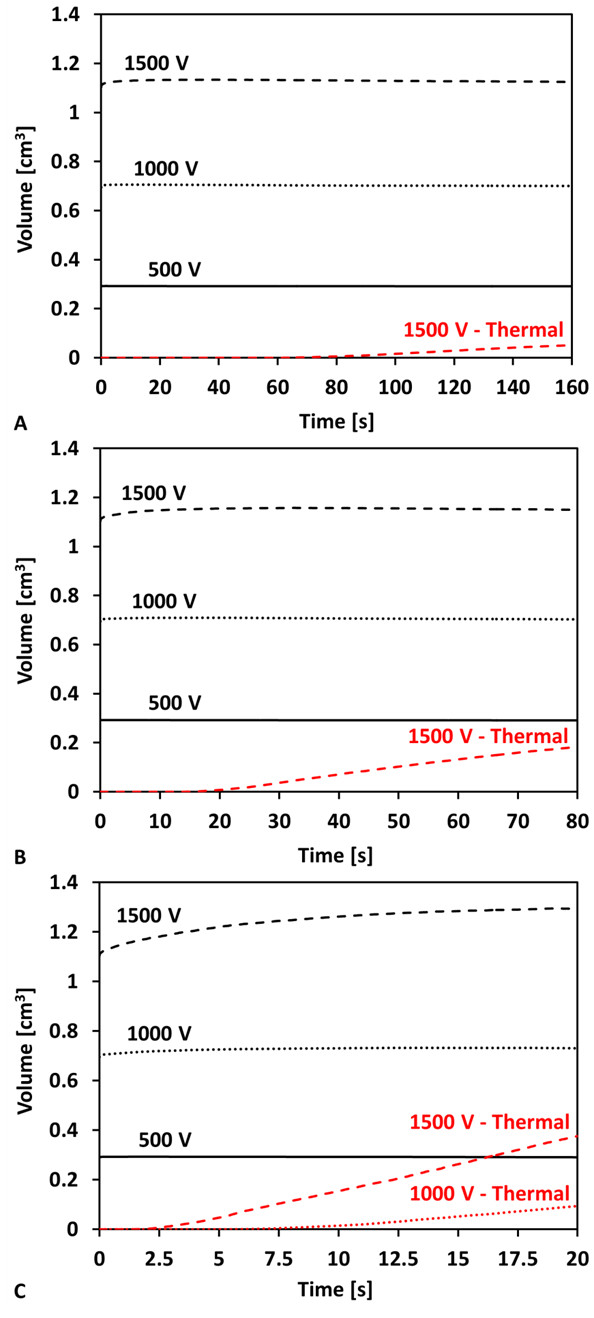
**Time history of the tissue volumes undergoing IRE alone or in superposition with thermal damage**. The IRE simulation used eighty pulses (50 μs) with frequencies of A) 0.5 Hz (160 s), B) 1 Hz (80 s), and C) 4 Hz (20 s). The applied voltages were 500 V, 1000 V, and 1500 V for each frequency investigated.

Although the volumes of tissue exposed to a minimum temperature can provide insight to the thermal effects resulting from a particular IRE protocol, they do not provide a quantitative measure of thermal damage based on established metrics in the literature [4,15,44,45]. In Figure 9 we provide plots that show the time dependence of the volume of tissue exposed to a minimum electric field of 500 V/cm, which was found to be the IRE threshold in grey matter for similar pulse parameters to those used in this study [22]. Additionally, we present the volume of tissue that undergoes thermal damage using the Arrhenius analysis presented in the methods section. Similar to the previous analysis, in Figure 9 we investigate the influence of increasing the frequency of pulse delivery in both predicted IRE treatment and thermal damage volumes. Specifically, the curves displayed in Figure 9 correspond to the IRE treated volumes with 500 V (0.293 cm^3^), 1000 V (0.706 - 0.732 cm^3^), and 1500 V (1.134 - 1.296 cm^3^).

An IRE treatment using applied voltages of 500 V and 1000 V did not result in any thermal damage when delivered at 0.5 Hz. For these cases there was sufficient time for the heat to dissipate through conduction and blood perfusion prior to the onset of the following pulse. However, 1500 V pulses delivered at 0.5 Hz resulted in thermal damage in 0.052 cm^3 ^(4.6%) of the IRE treated tissue. In Figure 9B, there are virtually identical IRE treatment volumes for the 1 Hz repetition rate as the 0.5 Hz of Figure 9A, but when applying 1500 V, there is some thermal damage generated within 20 seconds that affects about 0.183 cm^3 ^of tissue, approximately 16% of the IRE volume. Finally, Figure 9C displays the IRE and thermal damage volumes for the 4 Hz treatment. In this case, thermal damage occurs in 0.094 cm^3 ^(12.8%) of the tissue when applying 1000 V, and approximately 29% of the IRE volume is thermally damaged (0.376 cm^3^) by increasing the voltage to 1500 V. Additionally, if one focuses on the first seconds of the 1500 V, there is also an increase in the IRE lesion volume due to the increase in the temperature, and thus the electric conductivity.

## Discussion

We previously reported on the first experience applying IRE to the deep subcortical white matter of canine brain [23]. In that procedure electrodes were placed under CT-guidance through minimally invasive 1.2 mm diameter burr holes in order to produce a lesion. Temperatures were measured during this procedure, including the location in close proximity to where the lesion was produced. The low temperatures measured by our system confirmed the unique, non-thermal mode of IRE cell death. The *ex vivo *lesion volume was smaller than that observed from the *in vivo *MRIs due to elimination of edema as well as brain shrinkage during the fixation process. There was also limited time for the lesion to evolve relative to our previous work since the experimental aim of this study was to perform the procedure deep in the brain and evaluate the thermal effects, and therefore did not include the 3-day survival [21].

The ability of IRE to focally ablate small volumes of brain tissue in a minimally invasive fashion has significant potential clinical implications for the treatment of brain diseases in which destruction of focal neuroanatomic target is desired, such as some forms of epilepsy or central neuropathic pain syndromes [54]. We have shown in previous studies the ability to safely produce lesions in the grey matter of the brain cortex [21,22]. However, many of the potential central nervous system targets may reside deep within the brain, including the white matter [54]. Therefore, it is important to show the ability of IRE to produce a lesion deep within the white matter of the brain. To the best of our knowledge, we performed the first report of a CT-guided intracranial IRE treatment, as well as the first showing IRE pulses may be delivered within the deep white matter of the brain without causing significant edema [23]. We believe that the rapid implementation, minimally invasive nature, and precision offered by image guided IRE will be the preferred treatment delivery platform for future applications of this technology in the brain.

It should be noted that the thermal effects are most prevalent closest to the electrodes, where the electric field magnitude is also highest. Therefore, any thermal damage induced by an IRE procedure should occur within the targeted ablation volume and will not eliminate the effectiveness of the treatment. However, IREs unique non-thermal mechanisms are the key to its ability to be implemented in the vicinity of sensitive structures such as blood vessels and major nerves, a major limitation to resection and thermal therapies. Therefore, a comprehensive and accurate understanding of the potential thermal effects and/or damage is essential to ensure maintaining of these advantages and mitigating the challenges associated with thermal therapies.

Based on the electrodes configuration, measured electrical current and temperature in one canine, we developed a parametric study to investigate the effect of pulse frequency on three different applied voltages of 500, 1000, and 1500 V. The parametric study provides a reliable method to develop treatment protocols to ensure the IRE protocol achieves localized cell death independently from thermal damage. The study was based on pulse frequency, and confirms that if pulses are delivered too rapidly, thermal damage ensues and many of the benefits from this technology will not be optimized for the patient treatment.

The described method of this study takes pulse parameters (frequency, magnitude, and number of pulses) into account in addition to the dynamic changes in tissue electrical conductivity due to temperature increase as well as electroporation. Furthermore, the model accounts for the biological processes of the Pennes' Bioheat Equation, including metabolic heat generation and blood perfusion. Several researchers have demonstrated that blood perfusion is compromised after electroporation in organs outside the central nervous system, thus the heat dissipation from blood convection will be reduced, and it becomes even more important to decrease the frequency of pulse delivery in order to allow for thorough heat dissipation through conduction [55-57]. Even though the effects of pulse duration, electrode exposure, and separation distance were not explicitly investigated in this manuscript, the method can be readily adapted in order to select protocols that do not generate thermal damage in superposition with IRE. Thus, it is necessary that models are explored for each particular application in order to optimize the treatment protocol and better predict the treatment outcome.

Several values have been reported in the literature describing the amount that the electric conductivity of brain tissue increases per degree Celsius [39,58,59]. To be conservative, we selected 3.2%°C^-1 ^as the temperature coefficient in this study as reported by Duck *et al. *[39]. This value is higher than other reported values in the literature that range between 1.4 - 2.0%°C^-1 ^[58,59], resulting in higher calculated temperatures for our models. In order to assess the effect of a lower temperature coefficient, we simulated treatments with a 1.6%°C^-1 ^value since it is half of the magnitude used in the parametric study and it is still within the range reported in the literature. The volumes of tissue treated with IRE at the onset of the pulses were identical to the ones reported in Table 3 with the dynamic function. At the completion of the pulses, the 1000 and 1500 V applied voltages resulted in smaller volumes of tissue treated with IRE compared to the values reported in Table 3. As with the 3.2%°C^-1 ^temperature coefficient, there were no significant increases in the predicted IRE treatment volume for the 500 V trials. Applying 1000 and 1500 V resulted in IRE treatment volume increases of 1.26 - 3.27% and 1.91 - 8.85% versus those calculated at the onset of the pulses, respectively (before thermal effects begin). The increase in electric conductivity and thus IRE treatment volumes due to the thermal effects are moderate compared to the effects of electroporation. Nevertheless, the electric conductivity dependence on temperature must be incorporated from a thermal perspective in order to optimize IRE protocols, while minimizing any potential thermal damage. IRE is an emerging focal ablation technique and it is vital for researchers and physicians to work together in developing the numerical models for predictable treatment planning. The models presented here provide insight to the role of electroporation and temperature in the resulting volumes of tissue ablated with IRE alone or in superposition with thermal damage. The aim of this work was to provide the reader with numerical methods capable of evaluating pulse parameters used clinically to maximize the benefits of a non-thermal mode of tissue ablation. The numerical methods presented are capable of delineating volumes of tissue undergoing IRE from volumes undergoing thermal damage as a function of time. In this manner, the time point at which different treatment protocols achieve IRE while preventing thermal damage can be determined. It is important for researchers and physicians to be aware of the upper limit of IRE in order to maximize the benefits of a non-thermal model of tissue ablation. Future work should correlate the electric field distribution from these numerical models with reconstructed IRE lesions as seen in MRI and histopathology in order to generate an electric field threshold for brain tissue for clinical use. Future investigations should also determine the electrical magnitudes at which the increase in electrical conductivity occurs for grey matter, white matter, and pathologic brain tissue.

## Conclusion

We present the results of a parametric study in brain tissue that investigates 3 voltages delivered at 3 different frequencies which have been used clinically in other tissues such as prostate, kidney, and lungs [24-27]. These numerical simulations were based on an *in vivo *experimental procedure where a lesion was produced in the white matter of brain [23]. The procedure was performed in a minimally invasive fashion through 1.2 mm diameter burr holes and electrode placement was confirmed with CT imaging [23]. For the first time, the current and temperature were measured together in real-time during the delivery of the pulses and were used together as the basis for the numerical models. The models included all relevant pulse parameters and dynamic changes during treatment, and were capable of determining whether the lesions occurred due to IRE alone or in superposition with thermal damage. IRE alone allows preservation of the major vasculature, extracellular matrix and other critical structures, while achieving cell death in a target location. We hope our results provide physicians and researchers a way to assess individual protocols in order to capitalize on the benefits of this non-thermal mode of tissue ablation.

## Competing interests

PAG, JHR, REN, and RVD have patents pending on this technology.

## Authors' contributions

PAG, JHR, REN, TLE, and RVD conceived and designed the experiments. PAG, JHR, REN, and RVD performed and analyzed the experiments and numerical modeling. PAG, JHR, REN, TLE, and RVD drafted and critically revised the manuscript. All authors read and approved the final manuscript.
